# The Red Cross

**Published:** 1900-05-12

**Authors:** 


### THE RED CROSS.

In a letter to a contemporary,[1] Sir William Thomson says that the present arrangements for displaying the Red Cross flag are quite insufficient to ensure that the nature of the tent to which it is attached shall be made clear to the enemy; and he considers that the practical invisibility of the flag is accountable for some of the cases in which the Boers have unwittingly, as he suggests, fired upon ambulances and hospitals, especially the latter. He says that under certain circumstances the Geneva Red Cross, displayed as it usually is upon a flag, is practically invisible, and he gives an instance. "The morning was hot and sultry. There was hardly a sign of motion in the air. I said to a distinguished officer, 'If this camp were attacked by the enemy now, how could they identify your hospital?' 'Oh,' he said, 'we have our Red Cross flags up.' I asked, 'Where?' He answered, 'There, and there, and there.' 'Oh, yes,' I said, 'they are up, but who could see them at a thousand yards off?' And I pointed out that the flags were lying motionless along the flagstaff in folds." He adds that he can quite understand how an enemy could send shell after shell into a hospital under the belief that he was attacking a combatant portion of the army, for an enemy might see only a group of tents precisely similar to those used by the fighting men, and if the wind were not sufficient to extend the flag there would be nothing to tell him that he was firing on a crowd of sick and wounded men. At the distance at which shots are now fired even the most careful search with a telescope would not enable anyone to identify the hospital. Sir William suggests that instead of flags being hung from a pole they should be stretched upon a frame of some sort, but at any rate that some better method than that at present in use should be employed for indicating the hospital buildings.

[1] Brit. Med. Jour., May 5.

